# Population Structure and Genetic Diversity in Korean Cowpea Germplasm Based on SNP Markers

**DOI:** 10.3390/plants9091190

**Published:** 2020-09-12

**Authors:** Eunju Seo, Kipoong Kim, Tae-Hwan Jun, Jinsil Choi, Seong-Hoon Kim, María Muñoz-Amatriaín, Hokeun Sun, Bo-Keun Ha

**Affiliations:** 1 Department of Applied Plant Science, Chonnam National University, Gwangju 61186, Korea; dmswn3562@gmail.com; 2 Department of Statistics, Pusan National University, Busan 46241, Korea; kkp7700@gmail.com; 3 Department of Plant Bioscience, Pusan National University, Busan 46241, Korea; thjun76@pusan.ac.kr; 4 Jeollanamdo Agricultural Research and Extension Services, Naju 58213, Korea; jinsil45@korea.kr; 5 National Agrobiodiversity Center, National Institute of Agricultural Sciences, RDA, Jeonju 54874, Korea; shkim0819@korea.kr; 6 Department of Soil and Crop Sciences, Colorado State University, Fort Collins, CO 80523, USA; Maria.Munoz_Amatriain@colostate.edu

**Keywords:** cowpea, genetic diversity, population structure, single-nucleotide polymorphism

## Abstract

Cowpea is one of the most essential legume crops providing inexpensive dietary protein and nutrients. The aim of this study was to understand the genetic diversity and population structure of global and Korean cowpea germplasms. A total of 384 cowpea accessions from 21 countries were genotyped with the Cowpea iSelect Consortium Array containing 51,128 single-nucleotide polymorphisms (SNPs). After SNP filtering, a genetic diversity study was carried out using 35,116 SNPs within 376 cowpea accessions, including 229 Korean accessions. Based on structure and principal component analysis, a total of 376 global accessions were divided into four major populations. Accessions in group 1 were from Asia and Europe, those in groups 2 and 4 were from Korea, and those in group 3 were from West Africa. In addition, 229 Korean accessions were divided into three major populations (Q1, Jeonra province; Q2, Gangwon province; Q3, a mixture of provinces). Additionally, the neighbor-joining tree indicated similar results. Further genetic diversity analysis within the global and Korean population groups indicated low heterozygosity, a low polymorphism information content, and a high inbreeding coefficient in the Korean cowpea accessions. The population structure analysis will provide useful knowledge to support the genetic potential of the cowpea breeding program, especially in Korea.

## 1. Introduction

Cowpea provides a cheap source of dietary protein and essential nutrients for the people in sub-Saharan Africa, East Asia, and other developing countries [1]. According to the FAO, cowpea is extensively grown by millions of African farmers and is a great source of essential food for many urban consumers. It contains high levels of carbohydrates (~64%), protein (~25%), vitamins, and minerals, such as iron, calcium, thiamin, and folic acid [2,3]. Aside from its suitability for human consumption, cowpea also has high nutritional value required for animal fodder [4,5]. Furthermore, cowpea is well adapted to heat and drought conditions, which will play an essential role in improving its resilience to climate change [1,6]. In addition, it is tolerant to low soil fertility because of its ability to fix nitrogen [6,7].

Cowpea is a highly self-pollinated crop and has a low level of genetic diversity because of several genetic bottlenecks during its domestication process [1,8]. Previously, cultivated cowpeas were classified into five different groups based on their flower color, pod length, pod color, and seed color [9,10]. However, depending on the growing region, cowpea has diverged into two main subspecies: the common cowpea or African cowpea (*Vigna unguiculata* L. Walp. ssp. unguiculata) and the asparagus bean or “yardlong” bean (*Vigna unguiculata* L. Walp. ssp. *sesquipedalis*) [4,11]. The *unguiculata* subspecies is grown primarily for seeds in sub-Saharan African regions. Many accessions display a “bush”-type plant architecture, have a determinate growth habit, and have a short pod length [12]. Moreover, its ripe pods have higher fiber content that is a great source for animal fodder. The *sesquipedalis* subspecies is grown for tender and crisp pods that are consumed as a vegetable in China and South and Southeast Asia. It has an indeterminate growth habit, climbing and twining stems, and very long pods (30–50 cm in length) [11,13,14].

Genetic diversity analyses have allowed some prediction of the origins of the domestication and possible dispersion routes of cultivated cowpea [15,16,17,18]. Northeastern Africa was proposed as the domestication origin of cowpea due to this region having the greatest genetic diversity in wild cowpea [1]. From a gene pool structure analysis of a total of 442 cowpea landraces from 56 countries, Huynh et al. [19] suggested that divergent domestication processes from the wild cowpea led to the formation of two major gene pools (i.e., West Africa and East Africa) in cultivated cowpea in Africa. Based on genetic similarity, cowpea from West Africa was presumed to have been moved to Europe and India, while cowpea from East Africa was supposed to have been introduced to America and India [5,18,19]. Because of the existence of two major gene pools and the adaptation to more humid environments, India is considered a subdomestication region of cultivated cowpea [5,19].

Molecular markers are a useful tool for genetics and plant breeding [20]; they can easily be used for genetic diversity studies, genetic mapping, gene cloning, and marker-assisted selection [5,16,21]. In particular, single-nucleotide polymorphism (SNP) markers play a central role in plant breeding because of the abundance and high-throughput detection formats in the genome [22,23]. Many modern SNP genotyping platform technologies have been developed, including array-based, genotyping-by-sequencing (GBS), and restriction site-associated DNA sequencing (RAD-seq) [24]. In cowpeas, Munoz-Amatriain et al. [25] conducted whole-genome sequencing of 37 cowpea accessions and developed a Cowpea iSelect Consortium Array (Illumina, Inc.) containing 51,128 SNPs. This SNP array has been applied in various cowpea genetic studies, including diversity analyses of cultivated cowpea accessions from West Africa [25] and the Iberian Peninsula [18], and identification of the quantitative trait loci (QTLs) for domestication-related traits [26] and pod length [12]. In addition, GBS has been used for SNP discovery in a population structure analysis, as well as in association analysis of low phosphorus tolerance and cowpea mosaic virus resistance in the USDA cowpea germplasm collection [5,27,28]. Furthermore, Pan et al. [29] constructed a high-density SNP linkage map using RAD-seq analysis with two parents and 168 F2:3 lines, and identified the QTLs associated with yield-related traits in cowpea. 

In Korea, cowpea varieties were introduced from China between the 9th and 14th centuries [30]. Unlike in some other countries, cowpea is one of the minor crops generally used as a dietary nutritional supplement in Korea [30]. Therefore, only a few studies have been conducted to create new cowpea varieties, and only four varieties have been developed so far. Since the cowpea breeding program in Korea is not as advanced as those for other crops, the available genetic resources for cowpea will be important in the development of new varieties [31]. Currently, the Agricultural Genetic Resources Center in Korea retains a total of 762 cowpea accessions, and research on cowpea has been consistently ongoing. Lee et al. [32] used 52 accessions of cowpea to evaluate the genetic diversity in Korean cowpea using simple sequence repeats (SSRs) and amplified fragment length polymorphism (AFLP) markers. In addition, Lee at al. [30] analyzed the genetic diversity of 492 Korean cowpea landrace accessions using six SSR markers. However, the total number of accessions and molecular markers is still insufficient to elucidate the genetic architecture of Korean cowpea germplasms.

The objective of the current study is to understand the genetic diversity and population structure in 384 cowpea accessions, including 229 accessions from Korea. This study not only clarifies the genetic relationship between each cowpea accession, but also helps to utilize the genetic resources in Korean cowpea breeding and other research programs more efficiently.

## 2. Results

### 2.1. SNP Analysis

A total of 384 cowpea accessions were genotyped using the Illumina Cowpea iSelect Consortium Array containing 51,128 SNPs. The same custom cluster file used in the work of Munoz-Amatriain et al. [25] was applied to call genotypes within GenomeStudio v.2.0 software (Illumina, Inc., San Diego, CA, USA), and on average, a 95% SNP call rate was obtained across all accessions. After the SNP data were filtered for a missing rate of >10%, a minor allele frequency (MAF) of <0.05, and heterozygous calls of >10%, the final remaining 35,116 SNP loci were used for genetic analysis. In addition, eight cowpea accessions containing a missing rate of >10% and heterozygous calls of >10% were removed in this study. Therefore, we used a total of 376 cowpea accessions collected from 21 countries, including 43 with unknown origin (Tables S1 and S2).

For genetic analysis, the data were divided into two sets: a total of 376 cowpea accessions (global accessions) and 229 Korean cowpea accessions (Korean accessions). The global genetic diversity indicated a mean MAF of 0.269 and a polymorphic information content (PIC) of 0.287, while the *F*-statistics (F_is_) indicated a higher inbreeding coefficient (Table 1 and Table S3). The observed heterozygosity (H_o_) values ranged from 0 to 0.113, with an overall mean of 0.002, while the expected heterozygosity (H_e_) values ranged from 0.045 to 0.502, with a mean of 0.254. The genetic diversity of the Korean cowpea accessions indicated an MAF of 0.254 and a PIC of 0.265, while the F_is_ indicated a higher inbreeding coefficient. The values for H_o_ ranged from 0 to 0.138, with an overall mean of 0.003, while the values for H_e_ ranged from 0 to 0.521, with a mean of 0.234 (Table 1 and Table S4). Overall, the genetic diversity was similar between the global and Korean cowpea accessions.

### 2.2. Population Structure in the Global Cowpea Germplasm

**Figure 1:**
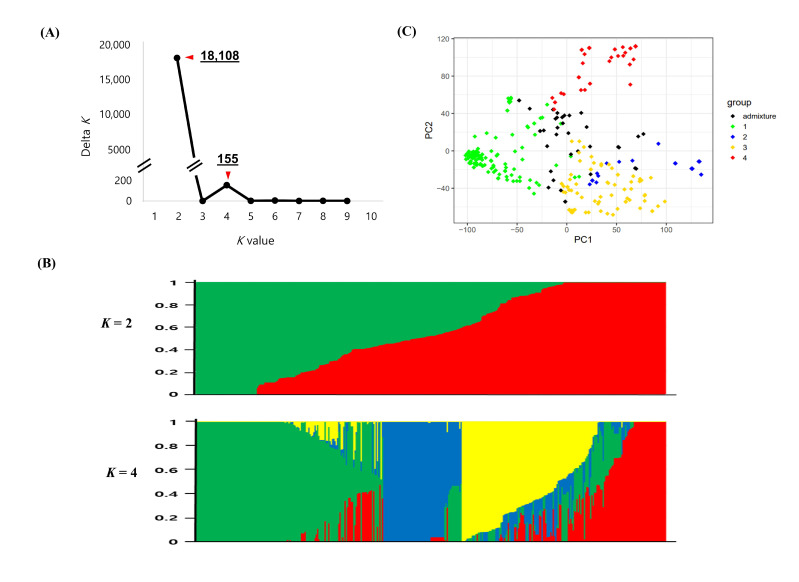
Population structure and principal component analysis (PCA) of the global cowpea accessions. (**A**) Delta *K* (Δ*K*) plot calculated from *K* = 2 to *K* = 9. (**B**) Population structure classification of the 376 global cowpea accessions using membership probability (Q-values). Each box plot within the different colored segments represents a different population. (**C**) PCA scatter plot of the 376 accessions along the PC1 and PC2 axes. The colors of the scatter plot are the same as those in the structure box plot.

**Figure 2:**
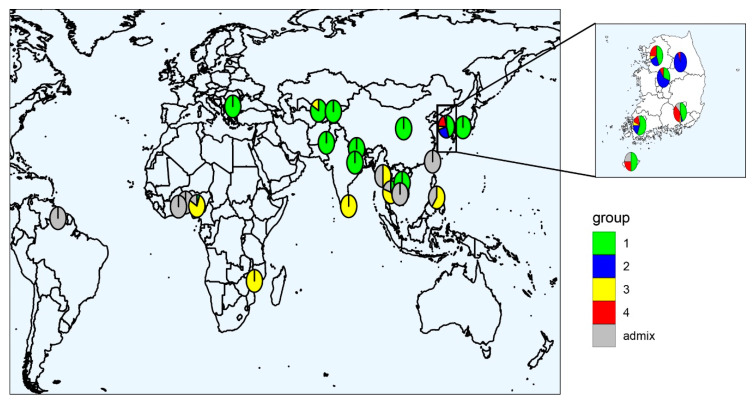
Geographical distribution of the global cowpea accessions showing the average proportion of the Q-values inferred by STRUCTURE (Q1, green; Q2, blue; Q3, yellow; Q4, red; admixture, gray).

The population structure of the 376 global cowpea accessions was inferred based on the 35,116 SNPs using STRUCTURE 2.3.4. The model-based structure analysis represented the delta *K* (∆*K*) peak (Figure 1a), showing two different peaks of ∆*K*, with the maximized peak observed at *K* = 2. This suggests that the 376 global cowpea accessions could be divided into two groups (Figure 1b). Using a likelihood threshold of 0.55, the 376 accessions were assigned to either group 1 (152) or group 2 (176); the remaining 48 accessions were a mixture of groups 1 and 2. The majority of the accessions in group 1 were from Korea (86) and Nigeria (28), and the majority of the accessions in group 2 were also from Korea (112) and Nigeria (23). In addition, the second peak of ∆*K* was observed at *K* = 4 (Figure 1a), indicating that the accessions could be further divided into four groups. Group 1 contained 150 accessions from Bulgaria, China, India, Japan, Korea, Kyrgyzstan, Nepal, Pakistan, Uzbekistan, Vietnam, Nigeria, and Thailand; group 2 contained 63 accessions from Korea; group 3 contained 77 accessions from Korea, Mozambique, Myanmar, Nigeria, the Philippines, Sri Lanka, Thailand, and Uzbekistan; and group 4 contained 49 accessions from Korea. Interestingly, groups 2 and 4 contained only Korean accessions. The 54 accessions from Nigeria were distributed into two groups, with 77.8% in group 3 and 5.6% in group 1. In addition, all seven Bulgarian accessions and five of the six Uzbekistani accessions were in group 1 (Table S5). The geographical locations of the global cowpea accessions were mapped based on the percentage of the Q-values obtained by STRUCTURE analysis (Figure 2). Without the Korean accessions, two larger groups were obtained (i.e., a high Q1 ratio group including Bulgaria (100%), China (100%), India (100%), Japan (100%), Kyrgyzstan (100%), Nepal (100%), Pakistan (100%), and Uzbekistan (83%); and a high Q3 ratio group consisting of Mozambique (100%), Nigeria (78%), the Philippines (60%), and Sri Lanka (100%)). The 229 accessions from Korea were distributed across all four groups, with 45% in group 1, 25.3% in group 2, 5.3% in group 3, and 20.1% in group 4 (Figure 2 and Table S5).

The results of the principal component analysis (PCA) agree with the model-based population structure analysis. Four clusters (Q1, green; Q2, blue; Q3, yellow; Q4, red) based on the STRUCTURE were obtained for the 376 cowpea accessions. The two-dimensional PCA plot explains 29.6% of the total genetic variance, containing 21.6% of the first principal component and 8.0% of the second principal component. Clusters 1, 3, and 4 were generally clustered separately, while some accessions of cluster 2 overlapped with those of cluster 3. This result might be caused by the larger introgression between clusters 2 and 3 or the lower power of a PCA using only two PCs compared with that of the STRUCTURE analysis. 

**Figure 3:**
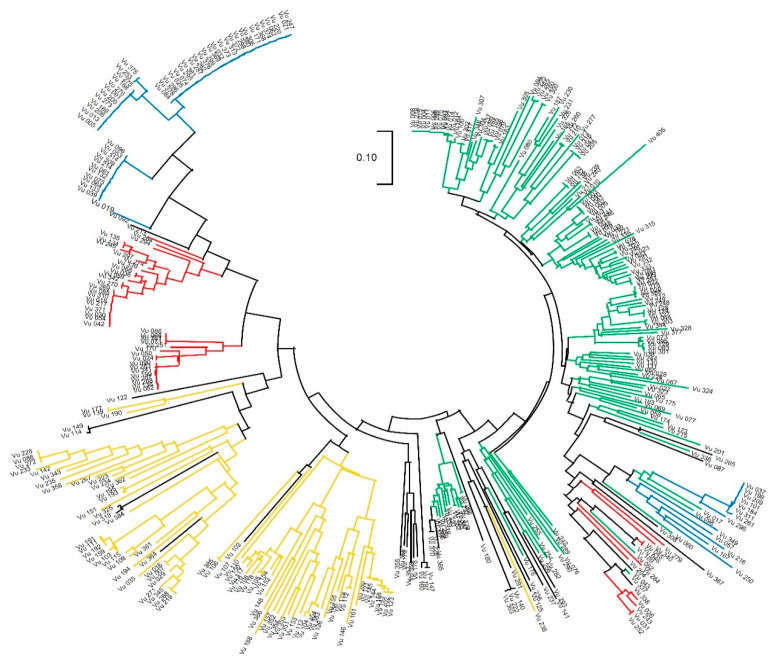
Neighbor-joining tree of the 376 global cowpea accessions using MEGA X, and the varieties in each colored branch are represented based on the structure population (Q1, green; Q2, blue; Q3, yellow; Q4, red; admixture, black).

The neighbor-joining tree analysis was calculated based on the genetic relationship and pairwise genetic distance between the 35,116 SNPs using the maximum likelihood method in MEGA X (Figure 3). The global populations were separated into four sections, namely, Q1 (green), Q2 (blue), Q3 (yellow), and Q4 (red), using the same colors as those in the structure analysis. The circular phylogenetic trees appeared to be largely divided into two categories: the first contained Q2 (blue), Q3 (yellow), and Q4 (red), while the second contained Q1 (green). In the first branch, Q3 (yellow) separated with Q2 (blue) and Q4 (red). Therefore, the results from the phylogenetic tree are consistent with the results from the population structure analysis.

### 2.3. Population Structure in the Korean Cowpea Accessions

**Figure 4:**
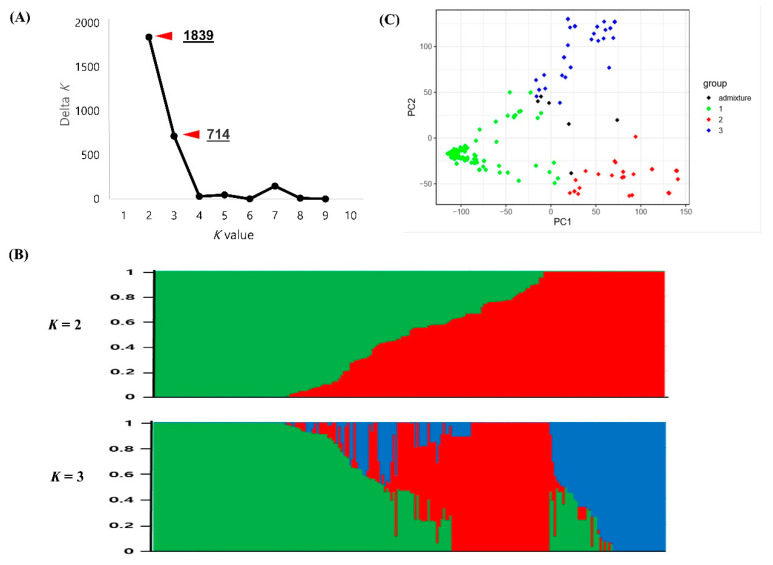
Population structure and principal component analysis (PCA) of the Korean cowpea accessions. (**A**) Delta *K* (Δ*K*) plot calculated from *K* = 2 to *K* = 9. (**B**) Population structure classification of the 229 Korean cowpea accessions using membership probability (Q-values). Each box plot within the different colored segments represents a different population. (**C**) PCA scatter plot of the 229 accessions along the PC1 and PC2 axes. The colors of the scatter plot are the same as those in the structure box plot.

The population structure of the 229 Korean cowpea accessions was also analyzed based on the 35,116 SNPs. Similar to that in the global cowpea accessions, the maximized peak was observed at *K* = 2. Group 1 contained 109 accessions, with most lines derived from Jeonra province (29) and Gyeongsang province (12). Group 2 contained 120 accessions, with many lines derived from Jeonra province (19) and Gangwon province (17). The second highest value of Δ*K* was at *K* = 3 (Figure 4a). Therefore, the Korean cowpea accessions were further divided into three groups. Group 1 contained 106 accessions, with most lines derived from the provinces of Jeonra (58%), Jeju (50%), and Gyeongsang (48%). Group 2 contained 70 accessions, with many lines derived from the provinces of Gangwon (94%) and Chungcheong (60%). Group 3 contained 47 accessions from the provinces of Gyeongsang (39%), Gyeonggi (25%), and Jeju (25%). Detailed information for each accession is listed in Table S6.

As expected, the PCA results from the 229 Korean cowpea accessions show three clusters (Q1, green; Q2, red; Q3, blue). The two-dimensional PCA plot explains 41.1% of the total genetic variance, containing 30.1% of the first principal component and 11.0% of the second principal component (Figure 4c).

**Figure 5:**
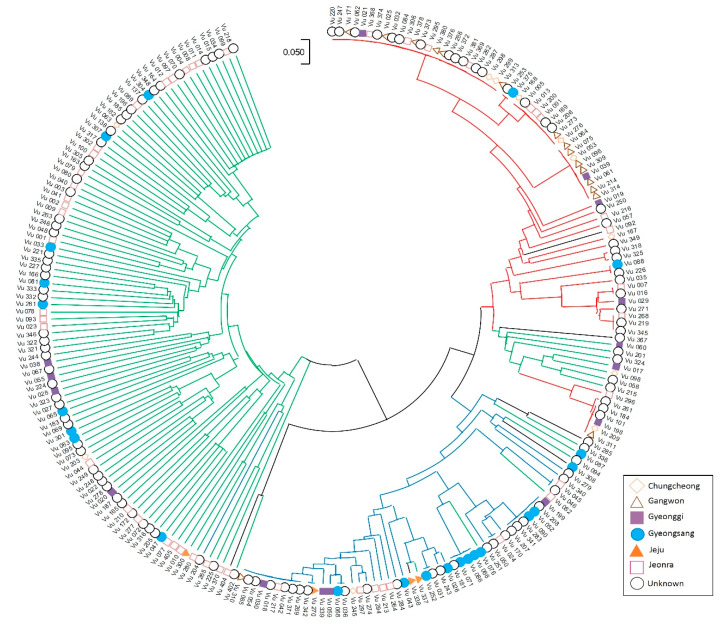
Neighbor-joining tree of the 229 Korean cowpea accessions using MEGA X. The varieties in each colored branch are represented based on the structure population (Q1, green; Q2, red; Q3, blue; admixture, black).

Similarly, the 229 Korean cowpea accessions showed three differentiated genetic populations containing admixtures, and the three structured populations are nearly clear (Figure 5). However, in detail, they were divided into two branches; the first contained Q1 (green) and the second included Q2 (red) and Q3 (blue). 

### 2.4. Analysis of Genetic Diversity

The genetic diversity statistics for the global and Korean cowpea accessions based on the structure analysis are listed in Table 2. For the global accessions, group 3 showed the highest level of genetic diversity, while group 2 the lowest. In the Korean cowpea accessions, group 2 showed the highest level of genetic diversity.

An analysis of molecular variance (AMOVA) was conducted to assess the population structure of both the global (376) and Korean (229) cowpea accessions based on the 35,116 SNPs. The AMOVA of the global populations shows that 41.5% of the total variation can be found between populations, while 58.5% between accessions (Table 3). In addition, the AMOVA of the Korean population shows that 47.5% of the total variation can be explained by the differences between the three populations, whereas 52.5% by the differences between accessions within populations (Table 3). Phi values of 0.4159 and 0.4747 in the global and Korean cowpea accessions indicate that the genetic differentiation can be found between groups.

## 3. Discussion

Analysis of the genetic relationship and diversity among plant accessions can lead to the sustainable use of genetic resources [18]. Previously, various studies were conducted to characterize the genetic diversity of 768 cowpea accessions from the USDA GRIN cowpea collection [5], wild and domesticated cowpea [1], and the general global cowpea cultivar [33]. In addition, Fang et al. [34] and Huynh et al. [19] distinguished the origin of cowpea as either West Africa, India, or Asia. However, there are few studies that have explored the genetic diversity of cowpea germplasm from Korea [30]. In this study, we investigated the genetic diversity and population structure in global and Korean cowpea accessions. The global cowpea accessions contained 376 accessions derived from 21 countries, including Korea and West Africa. We also separately analyzed 229 Korean cowpea accessions. The genetic diversity statistics of the global and Korean populations were analyzed using 35,116 high-quality SNPs.

Population structure analysis is very important for determining the genetic basis of complex traits for association analyses [35,36]. In this study, global cowpea accessions from 21 countries were dissected into two main populations, but individual accessions in groups were mixed without any correlation with geographical location. However, another peak at *K* = 4 suggested that the cowpea accessions could be further divided into four groups. The majority of the accessions in group 1 were from Asia and Europe, including Korea, China, Bulgaria, and Uzbekistan. The accessions in groups 2 and 4 consisted of only Korean accessions, while the majority of the accessions in group 3 were from West Africa, including Nigeria. In terms of geographical distribution, these four populations could be classified into three clusters: Accessions from Asia and Europe (group 1), accessions from Korea (groups 2 and 4), and accessions from Africa (group 3) (Figure 2). These results were consistent with the results of the PCA and phylogenic tree analysis. Similarly, the cowpeas from the worldwide collections were generally classified into three to four clusters. For instance, a total of 422 cowpea landraces collected from 56 countries were clustered into two distinct landrace groups (i.e., West Africa and East Africa) and one wild cowpea [19]. Moreover, a subset of the mini-core consisting of 298 lines from 50 countries was separated into three categories (i.e., Nigeria, Niger, and India) [37], whereas four major groups corresponding to East, Southern, and West Africa and Southern Europe gene pools occurred in 96 cowpea accessions containing the Iberian Peninsula cowpea [17]. Overall, the cowpea accessions from Asia and Europe are distinct from those from West Africa, as shown in this study. 

In addition to classification based on geographical origins, the global cowpea accessions were also categorized based on their agricultural traits, especially seed coat color. The accessions in group 1 were mainly black and cream in color, those in group 2 had a yellow seed coat, those in group 3 were brown and mixed in color, and those in group 4 were cream and mixed in color (Table S1). Notably, this is consistent with the fact that African cowpea accessions mainly have brown and speckled seed coats [38]. Conversely, Korean cowpea accessions had mostly cream and yellow seed coats in this study. Depending on how the cowpeas are consumed, countries or regions might have different preferences for the seed coat color [39]. Similarly, seed weight is an important agronomic trait that is significantly influenced by breeding programs. Groups 2 and 4 had a 100-seed weight value, which was larger than that of groups 1 and 3, showing that the Korean cowpea accessions have larger seeds than those from the other countries (Figure S1).

Cowpea is considered to have been domesticated in West and East Africa, and is presumed to have then been moved to Europe, Asia, and America [5,18,19]. In Korea, it has been speculated that cowpeas were introduced from China between the 9th and 14th centuries [30]. In the neighbor-joining tree analysis, the 229 Korean cowpea accessions were divided into Q1 (132; 57.6%), Q2 (46; 20.1%), Q3 (14; 6.1%), and Q4 (37; 16.2%) (Figure 3). More than 57% of the Korean cowpea accessions had a close genetic distance from the cowpeas from the European and Asian countries in Q1, which is consistent with the fact that Korean cowpeas were derived from China and Europe. The other Korean cowpea accessions were located in Q2 and Q4, which were on the same main branch as the West African accessions in Q3. It is estimated that frequent germplasm exchange between Korea and Africa might have occurred, and that the cowpea gene pools from Africa were introgressed or hybridized with the Korean cowpea gene pools. Overall, it is presumed that the Korean cowpea accessions were introduced directly from Africa or through Europe and Asia.

The Korean cowpea accessions were also dissected into two main populations, but individual accessions in groups were completely mixed for the global structure results. Another peak was observed at *K* = 3, suggesting the accessions were further dissected into three populations. The majority of the accessions in group 1 were from Jeonra and Gyeongsang provinces, those in group 2 were from Gangwon and Chungcheong provinces, and those in group 3 were from Gyeongsang and Gyeonggi provinces (Figure 4 and Figure 5). The Jeonra accessions were divided into three populations (58% in group 1, 23% in group 2, and 15% in group 3), while the Gyeongsang accessions were divided into two populations (48% in group 1 and 39% in group 2). Because cowpea in Korea has been cultivated mostly in Jeonra and Gyeongsang provinces, the transfer of these cowpea landraces might have occurred across the country through cowpea breeding activities. Therefore, the clustering was not strongly correlated with the collection sites from Jeonra and Gyeongsang provinces. Similarly, in a previous study, 492 Korean cowpea landrace accessions were poorly correlated with their collection sites [38]. However, in this study, 94% of the accessions from Gangwon province were clustered into group 2. In addition, 67% of the accessions in group 2 showed a yellow seed coat color. Meanwhile, the accessions in group 1 had black (48%) and cream (19%) seed coat colors, and those in group 3 had mixed (38%) and cream (34%) seed coat colors (Table S2). In addition, the accessions in groups 2 and 3 had larger seed sizes than those in group 1 (Figure S2). Because cowpea is mainly mixed with rice or used for rice cake stuffing in Korea, the seed size and seed coat color preferences could cause some genetic diversity in the Korean cowpea accessions. The human preference for lighter seed coat color during domestication has also been reported in peas [40] and chickpeas [38]. 

Cowpea is known as a highly self-pollinated crop, so a greater inbreeding coefficient of relationship was expected. For example, in a previous study by Fatokun et al. [37], 298 cowpea accessions showed a high F_is_ value (0.746). Herein, the genetic diversity analysis was conducted with 376 global and 229 Korean cowpea accessions, and both the global and Korean cowpea accessions in this study showed relatively high mean F_is_ values of 0.993 and 0.988, respectively (Table 1). Our higher inbreeding coefficient values might have been caused by the elimination of the eight accessions containing more than 10% heterozygous alleles. In addition, the average PIC (0.265) and He (0.234) values of the Korean cowpea accessions were similar to the values from a mini-core set from the global cowpea germplasm collection [37] and 96 cowpea accessions from 24 countries [17,18]. For the global accessions, the PIC values of Q1 and Q3 were higher than those of Q2 and Q4, which implies that Q1 and Q3 have more diverse accessions from different countries, which, in turn, might indicate a slightly higher genetic diversity (Table 2). As shown in the phylogenic tree (Figure 3), it could be considered that the genetic diversity in the Korean cowpea accessions (Q2 and Q4) was low, confirming the low genetic diversity in the Korean group as well (Table 2). However, in Korea, the genetic diversity of Q2 was slightly higher than that of the others, probably because of the high concentration of accessions in Gangwon province. However, the lowest genetic diversity among the Korean cowpea landraces was detected in Gangwon province [30]. This phenomenon might be because a previous study used different germplasm or a limited number of SSR markers. The cultivated cowpea tends to have a narrow genetic base due to its self-pollination mechanism, as well as the regional diversity of cowpea germplasm [6,21,41]. Furthermore, geographical or regional factors, such as temperature, humidity, and concentrations, could change the diversity of cowpea germplasm and their characteristics [7]. These results indicate that the Korean germplasm collections in this study were highly inbred and possessed normal levels of genetic diversity compared with those of other cultivated cowpeas.

## 4. Materials and Methods 

### 4.1. Plant Materials and Phenotypic Evaluation

A total of 384 cowpea germplasms were obtained from the Jeonnam Agricultural Research and Extension Services and the Rural Development Administration (RDA) Genebank at the National Agrobiodiversity Center, Republic of Korea. The accessions originated from the following countries: Benin (1), Bulgaria (7), Cambodia (1), Cote d’Ivoire (1), China (5), Ghana (1), Guyana (1), India (1), Japan (1), Korea (235), Kyrgyzstan (2), Mozambique (1), Myanmar (2), Nepal (3), Nigeria (55), Pakistan (1), the Philippines (5), Sri Lanka (2), Taiwan (1), Thailand (5), Uzbekistan (4), Vietnam (6), and unknown (43) (Table S1).

The cowpea accessions were planted in the experimental field of Chonnam National University on June 22, 2018, and June 19, 2019. Each accession was planted in a 1 × 1 m hill plot with one replication. After harvest, 100-seed weights were measured by weighting 100 seeds per accession. Seed coat color was classified into eight categories: (1) black, (2) brown, (3) purple, (4) pink, (5) light pink, (6) yellow, (7) cream, and (8) mixed color.

### 4.2. DNA Isolation and SNP Genotyping

Fresh leaves were sampled from each of the 384 germplasms. The genomic DNA was isolated from approximately 150–200 mg of leaf tissue from each accession using a CTAB-based method [42]. Electrophoresis gel and a NanoDrop 2000 spectrophotometer (Thermo Fisher Scientific, Waltham, MA, USA) were used to verify the DNA quality and concentrations. After DNA isolation, a total of 384 cowpea germplasms were genotyped with a 51K Cowpea iSelect Consortium Array at TNT Research Co. (Anyang, Gyeonggi-do, Republic of Korea) [43]. SNP calling was conducted using GenomeStudio v.2.0 software (Illumina, Inc.) with the same custom cluster file used in the study of Munoz-Amatriain et al. [25].

### 4.3. Filtering SNP Data

Raw data were filtered for quality control. First, SNPs containing missing data and heterozygous calls in >10% of the accessions and with a MAF of <5% were eliminated. Then, cowpea accessions with >10% missing SNP calls and heterozygous calls were eliminated using TASSEL 5.0 [44]. 

### 4.4. Population Structure Analysis

For the genetic diversity analysis, the filtered SNP data were divided into global and Korean groups. The former had a total of 376 germplasms, while the latter had 229 Korean accessions. The population structure was estimated using STRUCTURE v2.3.4 [45]. To estimate the number of populations (*K*), we set up a burn-in period of 10,000 iterations and a run length of 50,000 Monte Carlo Markov Chain (MCMC) iterations at each *K* value. Three independent runs were performed for each simulated value of *K*, ranging from 1 to 10. The optimal *K* value was determined using structure Harvester software [46]. For the proportional membership probability (Q), the probable cut-off for assignment to each cluster was ≥0.55 for three or more clusters. The box plots were “sort by Q” based on the optimal *K*. 

### 4.5. Principal Component Analysis (PCA) and Phylogeny Tree Analysis 

The two-dimensional PCA plot used the R built-in function “prcomp” to identify the structure in the distribution of genetic variation among geographical locations. The genetic diversity and relationships between the 376 cowpea germplasms and the genetic distance between accessions were calculated using 35,116 SNPs in MEGA X [47]. The phylogenetic trees were drawn based on the maximum likelihood tree method. The phylogeny test used the bootstrap method with 1000 bootstrap replications and the Tamura–Nei model, and the rates among sites were provided for an automatic initial tree (neighbor-joining). The tree color, shape, and branch of each genotype were drawn using the same color of the cluster (Q) from the STRUCTURE analysis. 

### 4.6. Genetic Diversity Data Analysis

The genetic diversity data analysis was conducted with the structure of the model-based program using the 35,116 filtered SNPs [48]. The genetic diversity statistics for the general total diversity, global *K* groups, and Korean *K* groups were calculated using the “hierfstat” R package [49]; these statistics included H_o_ (observed heterozygosity), H_e_ (expected heterozygosity), PIC (polymorphism information content), MAF (minor allele frequency), and F_is_ (*F*-statistics). The minimum, median, mean, and maximum were performed using Excel. To obtain the population differentiation, an AMOVA between and within groups in the global and Korean accessions was conducted using the “ade4” R package [50,51]. The statistical significance was tested with 999 permutations. Both the admixture and subpopulations were eliminated before the calculation.

## 5. Conclusions

A total of 35,116 high-density SNP markers were employed to estimate the genetic diversity in 376 cowpea accessions collected worldwide. These accessions were divided into four populations in the global group and three populations in the Korean group. In the global group, Q1 contained the Asian and European accessions, including Korean; Q2 and Q4 contained only Korean accessions; and Q3 contained the accessions from Nigeria. Based on the structure analysis and phylogenetic tree, the Korean cowpea accessions might have been introduced not only from Asia and Europe, but also directly from West Africa. In the Korean group, the 229 accessions were divided into three populations in which there was no strong correlation with the collection sites. However, group 2 contained mostly Gangwon accessions with yellow seed coat and large seeds. In addition, low genetic diversity was observed in the Korean accessions. These genetic structure and diversity analyses might help to identify beneficial alleles in Korean cowpea accessions through genomewide association studies in Korea.

## Figures and Tables

**Table 1:** The genetic diversity statistics of global (376 accessions) and Korean (229 accessions) cowpea accessions based on 35,116 single-nucleotide polymorphisms (SNPs).

	MAF	PIC	H_o_	H_e_	F_is_
**Global Accessions**					
Min	0.044	0.080	0.000	0.045	–0.091
Median	0.278	0.321	0.000	0.257	1.000
Max	0.500	0.375	0.113	0.502	1.000
Mean	0.269	0.287	0.002	0.254	0.993
**Korean** **Accessions**					
Min	0.000	0.000	0.000	0.000	–0.045
Median	0.263	0.313	0.000	0.246	1.000
Max	0.500	0.375	0.138	0.521	1.000
Mean	0.254	0.265	0.003	0.234	0.988

MAF, minor allele frequency; PIC, polymorphism information content; H_o_, observed heterozygosity; H_e_, expected heterozygosity; F_is_, *F*-statistics, inbreeding coefficient of an individual relative to the subpopulations.

**Table 2:** Genetic diversity statistics for the global optimal *K* groups and the Korean optimal *K* groups.

Category	H_o_	H_e_	F_is_	MAF	PIC
**Groups Based on Global Accessions**					
1	0.0016	0.2504	0.9889	0.1797	0.2027
2	0.0005	0.1421	0.9920	0.0904	0.1183
3	0.0018	0.3278	0.9928	0.2443	0.2580
4	0.0005	0.1895	0.9951	0.1328	0.1523
**Groups Based on Korean Accessions**					
1	0.0006	0.1888	0.9945	0.1355	0.1570
2	0.0017	0.2395	0.9887	0.1837	0.1939
3	0.0005	0.1903	0.9951	0.1460	0.1527

H_o_, observed heterozygosity; H_e_, expected heterozygosity; PIC, polymorphism information content; MAF, minor allele frequency; F_is_, *F*-statistics, inbreeding coefficient of an individual relative to the subpopulations.

**Table 3:** Analysis of molecular variance (AMOVA) among and within populations for cowpea accessions.

	Df	SS	MS	Sigma ^†^	Var. %	Phi ^‡^	*p*-Value
**Global Accessions**							
19Between pop	3	1,435,162	478,387.3	5965.9055	41.5	0.4159	<0.001
Between samples	335	2,806,352	8377.171	8377.1712	58.5		
Total	338	4,241,514	12,548.86	14,343.0768	100.0		
**Korean Accessions**							
Between pop	2	955,123.1	477,561.535	6761.786	47.5	0.4747	<0.001
Between samples	218	1,630,585.8	7479.751	7479.751	52.5		
Total	220	2,585,708.9	11,753.222	14,241.537	100.0		

^†^ Component of covariation; ^‡^ covariation differentiation statistic.

## Supplementary Materials

The following are available online at https://www.mdpi.com/2223-7747/9/9/1190/s1: Figure S1: Distribution of the 100-seed weight in the four different groups based on the population structure of the 376 global cowpea accessions; Figure S2: Distribution of the 100-seed weight in the three different groups based on the population structure of the 229 Korean cowpea accessions; Table S1: List of the global cowpea accessions and agronomic traits in this study; Table S2: List of the Korean cowpea accessions and agronomic traits in this study; Table S3: Raw data for the genetic diversity statistics of the global cowpea accessions; Table S4: Raw data for the genetic diversity statistics of the Korean cowpea accessions; Table S5: Population structure of the 376 global accessions based on 35,116 SNPs; Table S6: Population structure of the 229 Korean cowpea accessions based on 35,116 SNPs.
